# Methylation of *hMLH1* promoter correlates with the gene silencing with a region-specific manner in colorectal cancer

**DOI:** 10.1038/sj.bjc.6600148

**Published:** 2002-02-12

**Authors:** G Deng, E Peng, J Gum, J Terdiman, M Sleisenger, Y S Kim

**Affiliations:** Gastrointestinal Research Laboratory, 151M2 Veteran Affairs Medical Center, University of California, San Francisco, 4150 Clement Street, San Francisco, California, CA 94121, USA; Department of Medicine, University of California, San Francisco, 4150 Clement Street, San Francisco, California, CA 94121, USA

**Keywords:** colorectal cancer, *hMLH1*, DNA methylation, loss of heterozygosity

## Abstract

Microsatellite instability is present in over 80% of the hereditary non-polyposis colorectal carcinoma and about 15–20% of the sporadic cancer. Microsatellite instability is caused by the inactivation of the mismatch repair genes, such as primarily *hMLH1*, *hMSH2*. To study the mechanisms of the inactivation of mismatch repair genes in colorectal cancers, especially the region-specific methylation of *hMLH1* promoter and its correlation with gene expression, we analysed microsatellite instability, expression and methylation of *hMLH1* and loss of heterozygosity at *hMLH1* locus in these samples. Microsatellite instability was present in 17 of 71 primary tumours of colorectal cancer, including 14 of 39 (36%) mucinous cancer and three of 32 (9%) non-mucinous cancer. Loss of *hMLH1* and *hMSH2* expression was detected in nine and three of 16 microsatellite instability tumours respectively. Methylation at CpG sites in a proximal region of *hMLH1* promoter was detected in seven of nine tumours that showed no *hMLH1* expression, while no methylation was present in normal mucosa and tumours which express *hMLH1*. However, methylation in the distal region was observed in all tissues including normal mucosa and *hMLH1* expressing tumours. This observation indicates that methylation of *hMLH1* promoter plays an important role in microsatellite instability with a region-specific manner in colorectal cancer. Loss of heterozygosity at *hMLH1* locus was present in four of 17 cell lines and 16 of 54 tumours with normal hMLH1 status, while loss of heterozygosity was absent in all nine cell lines and nine tumours with abnormal *hMLH1* status (mutation or loss of expression), showing loss of heterozygosity is not frequently involved in the inactivation of *hMLH1* gene in sporadic colorectal cancer.

*British Journal of Cancer* (2002) **86**, 574–579. DOI: 10.1038/sj/bjc/6600148 www.bjcancer.com

© 2002 Cancer Research UK

## 

As an indicator of a distinctive carcinogenesis pathway, the phenotype of microsatellite instability (MSI) is present in over 80% of the hereditary non-polyposis colorectal carcinoma (HNPCC, Aaltonen *et al*, 1993) and approximately 15–20% of sporadic cancer (Thibodeau *et al*, 1993). Germline mutation of mismatch repair (MMR) genes (mainly *hMLH1* and *hMSH2*) are detected in over 70% of HNPCC patients with MSI (Kane *et al*, 1997; Papadopoulos and Lindblom, 1997). In tumours with MSI from patients with no obvious family history, mutations of MMR genes are rare. However, loss of *hMLH1* gene expression (gene silencing) was frequently observed in these tumours (Thibodeau *et al*, 1996). When methylation was analysed by *Hpa*II digestion method or methylation specific polymerase chain reaction (MSP) method, the methylation of CpG sites in the promoter region was generally consistent with the *hMLH1* silencing. However, there were many exceptions, where methylation was detected in normal expressing cells (Boyer *et al*, 1995; Cunningham *et al*, 1998; Herman *et al*, 1998; Veigl *et al*, 1998; Wheeler *et al*, 1999). In our previous study, we analysed the methylation status of the *hMLH1* promoter by using NaHSO_3_-sequencing method. We found that methylation in a more proximal region of the promoter (bases −248 to −178, relative to the transcription start site) was detected in all colorectal cancer cell lines which lacked gene expression, while in all expressing cell lines, the methylation was absent. However, methylation in a more distal region was present in all colorectal cancer cell lines, including the expressing cell lines (Deng *et al*, 1999). To examine whether this region-specific effect in cell lines also applies to primary tumours and gain a better understanding of the mechanism of the inactivation of MMR genes in sporadic colorectal cancer, we first evaluated a method, called ‘COBRA’ (Xiong and Laird, 1997), by comparing the methylation status of *hMLH1* promoter in cell lines determined using COBRA with the NaHSO_3_-sequencing method. We then used COBRA to analyse the proximal region and distal region of the *hMLH1* promoter in primary tumours with different expression levels. Since loss of heterozygosity (LOH) is one of the mechanisms for gene inactivation, and previous studies have detected LOH at the *hMLH1* locus in HNPCC tumours (Hemminki *et al*, 1994; Wheeler *et al*, 2000), it is important to know whether LOH mediates the inactivation of *hMLH1* in sporadic cancer. Therefore, we also analysed LOH at *hMLH1* locus in these primary colorectal cancers.

## MATERIALS AND METHODS

### Cell lines

Colorectal cancer cell lines SW1116, HCT8, Colo201, Colo320DM, CACO2, SW1463, HRT18, HT29, SW620, LS123, LS174T, HCT116, SW48, Lovo, and H498 were obtained from American Type Culture Collection (Manassas, VA, USA). Cell lines VACO5, VACO6, VACO411, VACO10P, VACO481 and VACO432 were kindly provided by Drs Sanford Markowitz and James KV Willson (Willson *et al*, 1987; Veigl *et al*, 1998). Cell lines RW2982 and RW7213 were from Dr Lance M Tibbetts. Cell lines C1a was derived from 5583s provided by Dr Fred T Bosman. Cell lines RKO and C were from Dr Michael Brattain. Cells were grown in DMEM supplemented with 10% foetal bovine serum at 37°C with 5% CO_2_ atmosphere.

### Tissues

Primary tumours were obtained at University of Chicago Hospitals, at Minneapolis Veteran Affairs Medical Center and at San Francisco Veteran Affairs Medical Center. All the 71 tumours were from patients with no known family history of colorectal cancer meeting the criteria of HNPCC, including 32 non-mucinous cancer and 39 mucinous cancer. Tumours were microdissected from formalin-fixed, paraffin-embedded histologic sections stained with haematoxylin-eosin as described previously (Deng *et al*, 1996). Normal mucosa of the same patient was also microdissected from the histological sections of normal tissue blocks taken at least 5 cm away from the tumours.

### MSI analysis

MSI of the primary tumours was determined by comparing the polymerase chain reaction (PCR) patterns of tumours with their normal counterparts amplified with the polymorphic loci BAT26, D3S2420, APC, D11S1999 and D18S877. Samples showing differences between normal and tumour in two or more loci were scored as MSI. Those showing no difference or difference in only one locus were scored as microsatellite stable (MSS).

### DNA methylation analysis

Methylation status of CpG sites in *hMLH1* promoter was determined by two methods based on the principles that cytidine in DNA is converted to thymidine when DNA is treated with NaHSO_3_, while methylated cytidine is protected from the conversion. Thus, the unmethylated and methylated cytidine can be distinguished by sequencing or digestion with a restriction enzyme, which recognizes a sequence containing CpG. The determination of methylation in *hMLH1* promoter with NaHSO_3_-sequencing method has been described previously (Deng *et al*, 1999). In the present study, we also used NaHSO_3_-digestion (or COBRA) method to analyse *hMLH1* methylation in cell lines and tumours (Xiong and Laird, 1997). To determine methylation in the proximal region of promoter, DNA was treated with NaHSO_3_, and amplified by PCR with primers 5′-TTTTGGTATTTTTGTTTTTATTGGT (upstream) and 5′-TCCAACCACCAAATAACCCCTA (downstream) covering the region from −322 to +56. PCR product was digested with restriction enzyme *Bst*UI which recognises CGCG sequence. After electrophoresis on a 2% agarose gel, the fraction of the digested fragments (92 base pairs (bp) and 286 bp) in the total of the digested and undigested fragment (378 bp) represented the per cent of methylation in the proximal region of *hMLH1* promoter. For methylation analysis of the proximal region in primary tumours, the similar procedure was carried out except that another downstream primer (5′-TAAAACAACTACTACCCACTACCTA) was used in PCR, and the undigested fragment (137 bp) and digested fragments (92 and 45 bp) were separated on a 6% polyacrylamide gel. To analyse methylation in the distal region, the NaHSO_3_ treated DNA was amplified by PCR with primers 5′-TTTTAGTTGTGATTTTTTAAGGTT (upstream) and 5′-AAAACAATAAAACCCTATACCTAA (downstream) covering the region from −796 to −547. The PCR product with 250 bp in length was digested with *Bst*UI, and electrophorised on a 2% agarose gel. The digested fragments (125, 67, 52 and 6 bp) represent the methylation in the distal region of *hMLH1* promoter.

### LOH analysis

LOH analysis was performed as described previously (Deng *et al*, 1996). LOH analysis at *hMLH1* locus in primary tumours was carried out by comparing the electrophoresis pattern of tumour and normal mucosa from the same patient after PCR with the primers D3S1768 and D3S2447, which are located at the same locus as *hMLH1* gene. The density of each band representing each allele was measured with a densitometer. The ratio of densities from two alleles in tumour sample was normalised by the ratio of densities from two alleles in normal sample. Tumours with the ratio of <0.5 or >2.0 were scored as LOH. The determination of LOH in cell lines was performed by analysing the patterns of each cell line after PCR with six di- and tetra-nucleotide polymorphism primers D3S2423, D3S2396, D3S1745, D3S1768, D3S2447 and D3S1611. Since these primers are located less than five centi-Morgans from *hMLH1* locus, and all of them show a high per cent of heterozygosity (from 0.58 to 0.87), it is highly unlikely (*P*=0.0007) that an individual will have two identical alleles in all six primers. Thus, sample with one single band after PCR with all these primers was scored as LOH (Wheeler *et al*, 1999).

### Immunohistochemistry analysis

The labelled avidin-biotin method was applied in the immunohistochemical staining as described (Thibodeau *et al*, 1996), using anti-*hMLH1* antibody (clone G168-15, PharMingen) and anti-*hMSH2* antibody (clone FE11, Calbiochem). After deparaffinisation, sections were subjected to heat-induced antigen retrieval in 10 mM sodium citrate buffer, pH 6.0 for 20 min. Nonspecific protein binding was blocked by incubating sections with 10% goat serum blocking solution (Zymed Histostain-Plus Kit) for 10 min. Anti-*hMLH1* was applied and incubated at 4°C overnight. Sections were rinsed in PBS followed by incubation of biotinylated secondary antibody (Zymed) for 10 min in room temperature. After a brief rinsing, streptavidin-enzyme conjugate (Zymed) was applied and incubated for 10 min. Sections were washed, followed by incubation with diaminobenzidine for 2–3 min. Without counterstaining, sections were dehydrated in graded ethanol and cleared in xylene. Staining for *hMSH2* was performed as above with the exception of antigen retrieval incubation for 10 min.

## RESULTS

### Loss of *hMLH1* expression is one of the main causes of MSI in colorectal cancer

In our previous study, by comparing MSI with the abnormality of MMR genes in colorectal cancer cell lines, we observed that alteration of *hMLH1* gene was the main cause of MSI, and the loss of expression was more frequently observed than mutations in *hMLH1* inactivation (Deng *et al*, 1999). In this study, we further analysed MSI in 71 primary colorectal cancers. MSI was detected in 17 cases (24%) of all sporadic cancers analysed ( Table 1

**Table 1:**
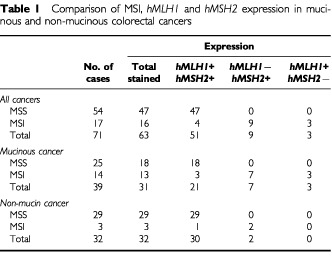
Comparison of MSI, *hMLH1* and *hMSH2* expression in mucinous and non-mucinous colorectal cancers

). To identify the inactivation of MMR genes, we measured *hMLH1* and *hMSH2* protein expression by immunohistochemical staining with anti-*hMLH1* and anti-*hMSH2* antibodies. Figure 1

**Figure 1:**
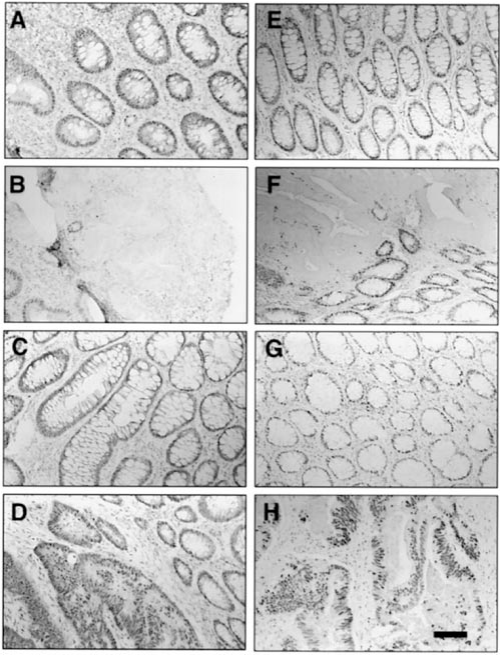
Immunohistochemical staining of colorectal cancers by *hMLH1*and *hMSH2* antibodies. Primary tumours (**B**, **D**, **F** and **H**) and their normal mucosa counterparts (**A**, **C**, **E** and **G**) from the same colorectal cancer patients were immunostained with anti-*hMLH1* antibody (**A** to **D**) or anti-*hMSH2* antibody (**E** to **H**) as described in Materials and Methods. Bar=160 μm.

shows the immunostaining of primary tumours (B, D, F and H) and their normal mucosa counterparts (A, C, E and G) from the same colorectal cancer patients with *hMLH1* antibody (A to D) and *hMSH2* antibody (E to H). Normal mucosa was positively stained with either *hMLH1* antibody (A and C) or *hMSH2* antibody (E and G). Tumours in B and F shows negative staining by *hMLH1* and *hMSH2* antibodies, respectively, while tumours in D and H were positively stained by these antibodies. We summarised the immunostaining data of cancers with MSS and MSI in Table 1. In the 47 stained cancers with MSS, all the samples were positively stained by both anti-*hMLH1* and anti-*hMSH2* antibodies. In the 16 stained tumours with MSI, nine showed negative staining with anti-*hMLH1* and positive staining with anti-*hMSH2*, and three showed positive with anti-*hMLH1* and negative with anti-*hMSH2*. The result indicated that lack of *hMLH1* or *hMSH2* protein expression is the cause of MSI in most tumours. However, there were four tumours with MSI that were positively stained with both anti-*hMLH1* and anti-*hMSH2* (Table 1). These tumours may have missense mutations in *hMLH1* or *hMSH2*, which destroy the normal function of DNA mismatch repair (Wheeler *et al*, 1999), or they may have mutations in other MMR genes. This hypothesis is true in both mucinous and non-mucinous cancers. MSI and *hMLH1*, *hMSH2* expression were compared in mucinous and non-mucinous cancer. MSI was present in 14 of 39 (36%) mucinous cancer and in only three of 32 (9%) non-mucinous cancer. Mucinous cancer shows significant higher incidence of MSI than non-mucinous cancer (*P*<0.01 by 2×2 chi square test). This observation indicated that mucinous cancer might have different pathogenic pathway compared with the non-mucinous cancer. However, we did not observe any significant difference in the *hMLH1* and *hMSH2* staining between mucinous and non-mucinous cancers with MSI. In 13 mucinous unstable cancers, seven and three cases showed negative *hMLH1* and *hMSH2* staining respectively. In three non-mucinous cancers with MSI, two cases were not stained with *hMLH1* antibody (Table 1).

### Methylation of CpG sites in a proximal region of *hMLH1* promoter, but not the distal region, is consistent with loss of expression in colorectal cancer

**Figure 2:**
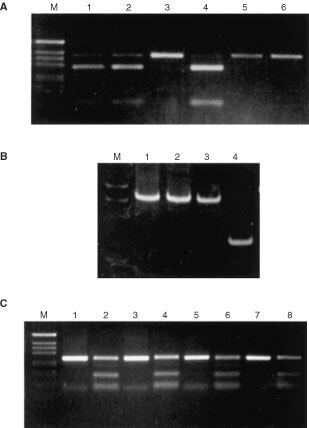
Methylation of *hMLH1* promoter determined by COBRA. (**A**) DNA of C1a, RKO, Lovo, VACO432, VACO481 and VACO457 (lanes 1–6, respectively) was treated with NaHSO_3_, amplified by PCR, digested with *Bst*UI, and separated on a 2% agarose gel. The ratio of the digested fragments (91 and 287 bp) to the total of the digested and undigested fragment (378 bp) represents the per cent of methylation at *Bst*UI site. The per cents of methylation in C1a, RKO, Lovo, VACO432, VACO481 and VACO457 are 95, 95, 0, 98, 0 and 0%, respectively. M, markers. (**B**) DNA from normal mucosa (lanes 1 and 3) and tumour (lanes 2 and 4) of two colorectal cancer patients C61 (lanes 1 and 2) and C64 (lanes 3 and 4) was used for methylation analysis in the proximal region. C61 tumour shows no methylation and C64 tumour shows complete methylation at *Bst*UI site in *hMLH1* promoter. M, markers. (**C**) DNA from normal mucosa (lanes 1, 2, 5, 6) and tumours (lanes 3, 4, 7, 8) of two colorectal cancer patients C61 (lanes 1–4) and C64 (lanes 5–8) was analysed for methylation in the distal region. The undigested products and *Bst*UI digested products are shown in lanes 1, 3, 5, 7 and lanes 2, 4, 6, 8, respectively. M, markers.

In the previous study, we have shown that methylation in a proximal region of *hMLH1* promoter invariably correlates with the absence of expression in colorectal cancer cell lines (Deng *et al*, 1999). To study whether this correlation still exists in primary tumour, we need to analyse the methylation status in primary tumours with different *hMLH1* expression levels. Thus, a simpler method for analysing methylation, COBRA, was evaluated by comparing methylation in colorectal cancer cell lines determined by this method ( Figure 2A

) with that by NaHSO_3_-sequencing (Deng *et al*, 1999). The methylation status determined by these two methods were identical. This is not surprising, since the *Bst*UI site used in COBRA is composed with two consecutive CpG sites within this proximal region. By using COBRA, we extended the methylation measurement to 64 primary tumours and mucosa from the same patients (Figure 2B). No methylation was detected in normal mucosa from these patients. In 55 anti-*hMLH1* antibody positive tumours, none showed methylation, while in nine anti-*hMLH1* antibody negative tumours, seven showed methylation. No methylation was found in the other two tumours with negative staining. These two tumours may represent those expressing a truncated protein (e.g. from nonsense, frameshift, or splice site mutations) which can not be recognised by the antibody that was used. In the previous study, we observed extensive methylation of CpG sites in Region A of *hMLH1* promoter in all colorectal cancer cell lines regardless of the RNA expression levels (Deng *et al*, 1999). To see whether this observation exists in primary tumours, we measured the methylation in Region A in the primary tumours and their normal counterparts (Figure 2C). Partial methylation (40–60%) was detected in all normal and tumour tissues tested, indicating that methylation in only a proximal region, but not in the distal region, correlates with the loss of expression, and that *hMLH1* silencing by methylation is region specific.

### *hMLH1* inactivation is mediated by biallelic methylation of the promoter or mutation of the gene rather than LOH

LOH at *hMLH1* locus was analysed in 26 cell lines. In 17 cell lines with normal *hMLH1* expression and wild type DNA sequence, four lines (SW1116, Colo201, RW7213 and H498) showed only a single band at each of the six consecutive polymorphic loci. We scored these four cell lines as LOH ( Table 2

**Table 2:**
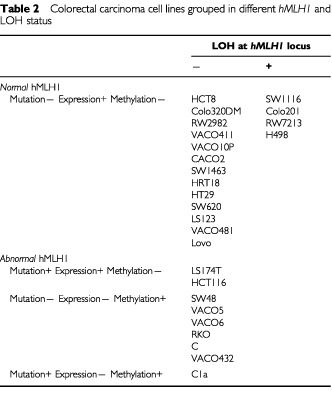
Colorectal carcinoma cell lines grouped in different *hMLH1* and LOH status

), because it is highly unlikely that these cells lines have two identical alleles in all six consecutive loci (see Materials and Methods section). In nine cell lines with abnormal *hMLH1*, including mutations and loss of expression, LOH was not detected. Since almost 100% methylation (Figure 2 and Deng *et al*, 1999) and non allelic loss was detected in seven cell lines with the silenced expression (SW48, VACO5, VACO6, RKO, C, VACO432 and C1a), it is reasonable to believe that both alleles of *hMLH1* promoter are methylated. This is consistent with the previous observation of biallelic methylation in VACO5 (Veigl *et al*, 1998). In the cell line C1a which shows both mutation and methylation, it is not clear which event occurs first. For the cells with mutations and normal expression, as in HCT116, the single truncated *hMLH1* protein band (due to the nonsense mutation at codon 252) was first explained by the loss of the wild type allele (Papadopoulos *et al*, 1994). However, it was reported later that HCT116 cell contained two intact chromosome 3 by karyotypic analysis (Koi *et al*, 1993). The LOH analysis in this study also proved that two *hMLH1* alleles were present in HCT116 (Table 2). Since DNA sequencing of HCT116 cell revealed sole and complete mutation at codon 252, we assumed that HCT116 contained two identical *hMLH1* mutant alleles. A similar possibility also applies to LS174T cell, since we only observed a complete missense mutation at codon 117 from reverse transcription–PCR product (Deng *et al*, 1999), and no LOH was detected at the *hMLH1* locus (Table 2). The missense mutation at this codon (codon 117) has been shown to alter normal *hMLH1* function by functional analysis (Shimodaira *et al*, 1998). Thus, the analysis of mutation and methylation in nine cell lines with abnormal *hMLH1* showed that loss of the normal function of *hMLH1* is mediated by either biallelic methylation in the promoter in six, by mutation and methylation in one, and by mutation in two cell lines. LOH at *hMLH1* locus in four of 17 cell lines with normal *hMLH1* status suggests that a tumour suppresser gene may be present adjacent to *hMLH1* locus, which is involved in colorectal carcinogenesis.

LOH status was also determined in primary tumours, and compared with the immunostaining by anti-*hMLH1* antibody ( Table 3

**Table 3:**
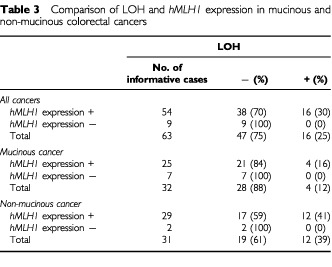
Comparison of LOH and *hMLH1* expression in mucinous and non-mucinous colorectal cancers

). In 54 tumours with positive staining, LOH was detected in 16 cases (30%), while in nine tumours with negative staining, no LOH was found. In the four cell lines (Table 2) and 16 primary tumours with LOH (Table 3), *hMLH1* expression was normal. This indicates that when one allele is deleted, the retained allele (wild type) can still perform normal function. However, some other tumour suppressor genes within this region may be inactivated by two hits, resulting in cancer formation. The observation that LOH is not present in primary tumours with negative *hMLH1* staining together with the similar observation in cell lines suggests that biallelic methylation or mutation, but not LOH, are the causes of the inactivation of *hMLH1* in cell lines and primary tumours of sporadic colorectal cancer patients with MSI. In the *hMLH1* positive staining tumours, LOH was present in four of 25 (16%) mucinous cancer and 12 of 29 (41%) non-mucinous cancer (Table 3).

## DISCUSSION

In previous studies, researchers measured methylation in two distal regions of *hMLH1* promoter by the methods of *Hpa*II digestion or methylation specific PCR. Even though methylation determined in these regions was generally consistent with the loss of gene expression, there were exceptions, and partial methylation was observed in some MSS cell lines with normal *hMLH1* expression (Deng *et al*, 1999; Wheeler *et al*, 1999). When we utilised NaHSO_3_-sequencing method to measure the methylation status in the whole *hMLH1*promoter region, we localised a proximal region in the promoter in which the methylation was invariably correlated to the loss of expression. We also observed that methylation was present in the distal region in all cell lines regardless of the expression level (Deng *et al*, 1999). Thus, in the present study, we decided to analyse methylation in both the proximal region and the distal region of *hMLH1* promoter in primary colorectal cancers. We used a simpler method (COBRA) to analyse methylation. Our results indicated that in primary tumours, methylation of CpG sites in the proximal region of *hMLH1* promoter is correlated with the loss of gene expression, while methylation is present in the distal region in all tissues tested, including normal mucosa and tumours which express *hMLH1*. The localization of methylation in *hMLH1* promoter could help to analyse methylation status more accurately in samples from cancer patients. For example, Markowitz *et al* detected methylation in the proximal region in three serum samples from nine colorectal cancer patients who had the same methylation in their tumours (Grady *et al*, 2001). This assay offered a potential mean for monitoring the treatment of colorectal cancer patients with MSI.

Elucidation of the mechanisms involved in *hMLH1* inactivation is important, since this knowledge may lead to the development of the diagnosis markers, or the ways for prevention and treatment of colorectal cancer with MSI. LOH is considered as one of the major mechanisms for the inactivation of tumour suppressor genes. In the present study, we did not find LOH in any cell lines or primary tumours (both mucinous and non-mucinous) with abnormal *hMLH1*, indicating that LOH is not a frequent mechanism of *hMLH1* inactivation in both cell lines and the primary tumours. The inactivation of *hMLH1* is mainly mediated through biallelic methylation of the promoter or mutation of the gene. However, LOH around *hMLH1* locus was detected in four of 17 cell lines (24%) and 16 of 54 primary tumours (30%) with normal *hMLH1*. This result suggests that another unidentified tumour suppressor gene or genes close to *hMLH1* gene may exist and play a role in colorectal carcinogenesis, especially in non-mucinous cancer with MSS phenotype. The significantly higher incidence of MSI in mucinous cancer as compared with non-mucinous cancer and higher incidence of LOH at chromosome 3p in non-mucinous cancer with MSS suggest different genetic aberrations and carcinogenesis in these two histological types of colorectal cancer.

In summary, our data shows that in sporadic colorectal cancers, methylation, but not LOH is frequently involved in *hMLH1* inactivation, and methylation correlates with *hMLH1* gene silencing in a region-specific manner. These observations are important not only for understanding the MSI pathway in colorectal carcinogenesis, but also has an important impact on the therapy of colorectal cancer, since drug resistance can be mediated by loss of mismatch repair, and recovering the mismatch repair function by demethylation might lead to the overcome of drug resistance (Moreland *et al*, 1999).
